# Anoikis-Related Gene Signature for Prognostication of Pancreatic Adenocarcinoma: A Multi-Omics Exploration and Verification Study

**DOI:** 10.3390/cancers15123146

**Published:** 2023-06-11

**Authors:** Jin Zhang, Xuesong Li, Yi Lu, Guowen Wang, Ying Ma

**Affiliations:** 1 Department of Bone and Soft Tissue Tumors, Tianjin Medical University Cancer Institute and Hospital, National Clinical Research Center for Cancer, Key Laboratory of Cancer Prevention and Therapy, Tianjin’s Clinical Research Center for Cancer, Tianjin 300060, China; zjbone123@tmu.edu.cn; 2 Department of Pancreatic Cancer, Tianjin Medical University Cancer Institute and Hospital, National Clinical Research Center for Cancer, Key Laboratory of Cancer Prevention and Therapy, Tianjin’s Clinical Research Center for Cancer, Tianjin 300060, China; lixuesong717@tmu.edu.cn (X.L.); luyi2021@tmu.edu.cn (Y.L.)

**Keywords:** anoikis, pancreatic adenocarcinoma, bioinformatics multi-omics, prognosis

## Abstract

**Simple Summary:**

Pancreatic cancer is one of the deadliest forms of cancer, with low survival rates and limited treatment options. Anoikis resistance, or the ability of cancer cells to survive and grow in the absence of attachment to the extracellular matrix, is thought to play a critical role in pancreatic cancer progression and treatment resistance. In this study, a multi-omics approach was used to identify a set of signature genes associated with anoikis in pancreatic cancer. The approach involved integrating data from large-scale cancer genomics databases such as TCGA and GEO, as well as single cell sequencing databases and in vitro assays. The identified signature genes have the potential to serve as prognostic biomarkers, allowing clinicians to predict patient outcomes and tailor treatment strategies accordingly. Additionally, these genes may also predict the chemo-sensitivity of pancreatic cancer cells, helping to guide the selection of chemotherapy regimens. The use of a comprehensive multi-omics approach provides a complete understanding of the molecular mechanisms underlying anoikis resistance in pancreatic cancer. This study highlights the importance of integrating multiple data sources and experimental approaches in cancer research to facilitate the discovery of new biomarkers and therapeutic targets. Such approaches have the potential to improve patient outcomes and lead to more effective cancer treatments.

**Abstract:**

Anoikis, a form of apoptosis that occurs due to detachment of cells from the extracellular matrix, has been linked to the development of cancer in several studies. However, its role in pancreatic cancer remains incompletely understood. In this study, we utilized univariate Cox regression and LASSO regression analyses to establish a prognostic model for pancreatic adenocarcinoma based on anoikis-related genes in the TCGA database. Additionally, we performed univariate and multifactorial Cox analyses of protein expression results for TCGA pancreatic adenocarcinoma. We further explored the difference in immune infiltration between the high-risk and low-risk groups and verified the expression of the screened genes using quantitative real-time PCR (qRT-PCR). Our findings indicate that numerous anoikis-related genes are linked to pancreatic adenocarcinoma prognosis. We identified seven prognostic genes (MET, DYNLL2, CDK1, TNFSF10, PIP5K1C, MSLN, GKN1) and validated that their related proteins, such as EGFR and MMP2, have a significant impact on the prognosis of pancreatic adenocarcinoma. Based on clustering analyses of the seven prognostic genes, patients could be classified into three distinct categories, for which somatic mutations varied significantly across the groups. High-risk and low-risk groups also exhibited significant differences in immune infiltration. All genes were found to be highly expressed in pancreatic cancer cell lines (ASPC-1, CFPAC-1) as compared to a normal pancreatic cell line (HPDE). Based on the seven anoikis-related genes, we formulated a robust prognostic model with high predictive accuracy. We also identified the significant impact of KRAS, P53, and CDKN2A mutations on the prognosis of this fatal disease. Therefore, our study highlights the crucial role of anoikis in the development of the pancreatic adenocarcinoma tumor microenvironment.

## 1. Introduction

Pancreatic adenocarcinoma (PAAD) is associated with a dismal prognosis, with a 5-year survival rate of only 2–9% [1]. Early clinical manifestations of PAAD are often absent, precluding timely surgical intervention upon diagnosis. Conventional therapeutic approaches, including radiotherapy, chemotherapy, and available targeted therapies, prove insufficient in most cases [2,3]. Thus, the identification of novel therapeutic targets and the establishment of a prognostic model capable of appraising patients’ outcomes to guide clinical treatment decisions are urgently required.

Anoikis, a special form of apoptosis triggered by the detachment of cells from the extracellular matrix (ECM), curbs the metastatic potential of tumor cells that disseminate while carrying the ECM. Anoikis safeguards against the aberrant proliferation of detached tumor cells, distal metastasis, and thus occupies a pivotal position in inhibiting tumor progression [4]. The involvement of anoikis-related genes in tumorigenesis and tumor metastasis has been widely reported in various cancer types, including gastric, breast, lung, and endometrial cancers [5,6,7,8].

Although numerous molecular biology studies have unveiled the impact of anoikis on pancreatic adenocarcinoma, most of these studies have scrutinized individual genes and mirRNA, making it arduous to illustrate the overarching influence of anoikis in pancreatic adenocarcinoma development [9,10,11].

Hence, our research aimed to leverage transcriptomics and proteomics analyses to demonstrate the significant role of anoikis in pancreatic adenocarcinoma development and to scrutinize somatic mutations in pancreatic adenocarcinoma. We intend to establish a prognostic model that evaluates the prognoses of clinical pancreatic adenocarcinoma patients and explores novel targets for pancreatic adenocarcinoma therapy.

## 2. Materials and Methods

### 2.1. Data Source

We utilized data from the UCSC Xena and TCGA official website to investigate the transcriptome, somatic mutations, and proteomic information in pancreatic adenocarcinoma. We excluded patients with paraneoplasia and a lack of survival time, ultimately using transcriptome information for 176 patients and somatic mutation information for 169 patients. Additionally, we obtained proteomic information for 110 pancreatic adenocarcinoma patients. To validate our findings, we used transcriptomic information from 63 patients from GSE57495 as a validation group. We selected a total of 445 genes associated with anoikis from GeneCards, focusing on genes with correlation scores >0.4.

### 2.2. Construction and Validation of a Risk-Scoring Model with Transcriptomic Data

We sought to identify crucial genes linked to prognosis using transcriptomic data obtained from TCGA. We employed a two-step approach, beginning with univariate Cox analysis to detect potential candidates. Subsequently, we implemented LASSO regression analysis to further narrow down our list to include only genes associated with anoikis. Our risk score, calculated on the basis of the expression and coefficient for each gene (risk score = (coefi × expri)), permitted the classification of TCGA patients as high- or low-risk groups depending on the median risk score. To evaluate the accuracy of our risk score, we employed additional data from GSE57495.

### 2.3. Proteomics Analysis for Further Validation

We screened prognostic proteins via univariate Cox analysis for the proteomic information of TCGA and performed a multifactorial Cox analysis together with our constructed risk scores.

### 2.4. Clustering and Analysis of Somatic Mutations

We performed a cluster analysis based on the prognostic genes of the risk score model. The clustered groupings were also analyzed for different somatic mutations.

### 2.5. Immune Infiltration in the Low-Risk and High-Risk Groups

The CIBERSORT algorithm was used to calculate the abundance of 22 kinds of immune cells in the low-risk and high-risk groups. We also carried out ssGSEA analysis (single sample Gene Set Enrichment Analysis) in the low-risk and high-risk groups to compute the contents of 28 kinds of immune cells. Finally, the differences in PDCD1, CD274, PDCD1LG2, CTLA4, CD80, and CD86 expression between the low-risk and the high-risk groups were compared.

### 2.6. Enrichment Analysis

We screened the differential genes between the high-risk and low-risk groups, and analyzed the enrichment of these genes through Gene Set Enrichment Analyses (GSEA), Gene Ontology (GO) analyses, and the Kyoto Encyclopedia of Genes and Genomes (KEGG) analyses. The differential gene screening multiple was set to 1.

### 2.7. Validation in Single Cell Sequencing

We downloaded raw data for single-cell sequencing from published single-cell sequencing data for pancreatic adenocarcinoma [12]. Principal component analysis (PCA) and Uniform Manifold Approximation and Projection (UMAP) algorithms on single-cell sequencing samples were carried out. The expressed cells were clustered using the “Seurat” package in R software 4.1.3. The “SingleR” package and typical cell marker genes were used for annotation.

### 2.8. Drug Sensitivity Analysis

Determine which drugs have different sensitivities in the low- and high-risk groups to screen for potential therapeutic agents for pancreatic adenocarcinoma in our prognostic model. We performed drug sensitivity analysis using the limma, ggpubr, and pRRophetic packages in R language, with *p* < 0.001 as the screening criterion.

### 2.9. Cell Culture and Real-Time Quantitative PCR

Pancreatic adenocarcinoma cell lines (ASPC-1 and CFPAC-1) and normal pancreatic cells (HPDE) were cultured in a 37 °C incubator with 5% CO_2_ in the presence of 1% penicillin/streptomycin and 10% fetal bovine serum. The RNA from cell lines was extracted using TRIZOI reagent (Invitrogen, Carlsbad, CA, USA), according to the instructions for use. Following reverse transcription into cDNA, the samples were tested via qRT-PCR assay using a LightCycler 480 fluorescent system. The primer sequences in our study are listed in Table S1.

### 2.10. Statistical Analysis

We assessed correlations between continuous variables with Pearson analysis. We used *t*-tests for statistical analysis between the two groups, and analysis of variance for multi-group tests. A *p*-value < 0.05 was used to represent statistically significant results. All the statistical analysis was implemented with R software (R version 4.2.0) (* *p* < 0.05, ** *p* < 0.01, *** *p* < 0.001).

## 3. Results

### 3.1. Constructing a Risk-Scoring Model Using Anoikis-Related Genes

**Figure 1:**
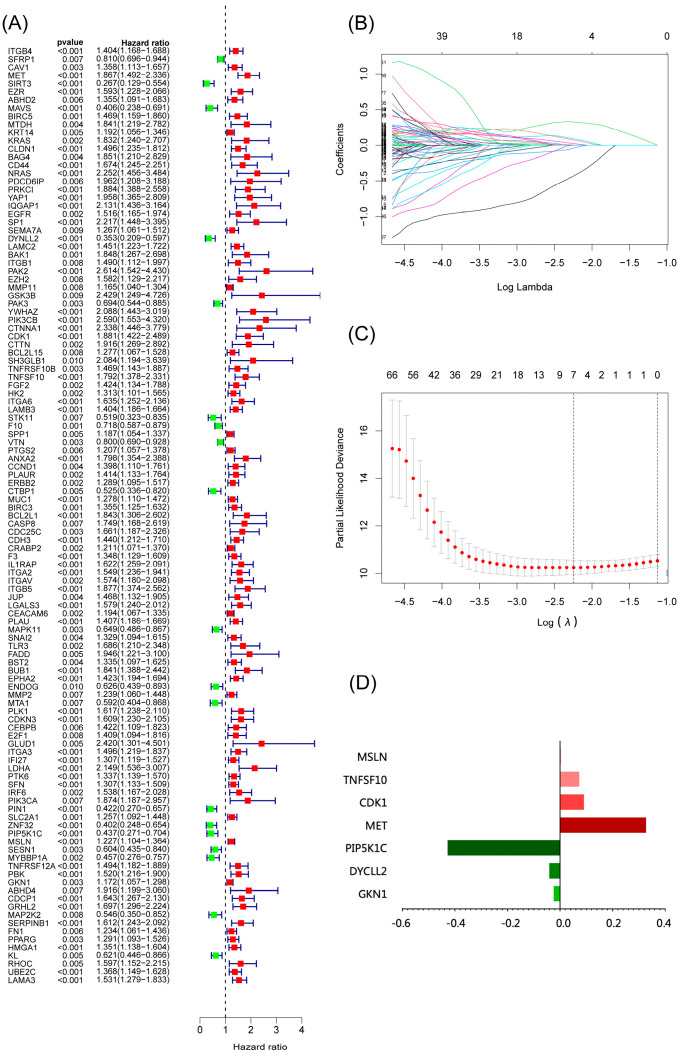
Construction of a risk-scoring model for patients with pancreatic adenocarcinoma based on the TCGA database. (**A**) Univariate Cox analysis demonstrated the correlation between anoikis-associated genes and prognosis. (**B**,**C**) LASSO regression analysis further screened the prognostic genes of B cell markers. (**D**) Score composition of riskscore.

We used anoikis-related genes to build a risk model for predicting prognosis from the TCGA database and validated the model in the GEO dataset following our flow chart in Figure S3. By univariate Cox analysis, we identified 115 anoikis-related genes associated with prognosis (Figure 1A). Seven anoikis-related genes were screened by LASSO regression analysis (MET, DYNLL2, CDK1, TNFSF10, PIP5K1C, MSLN, GKN1) (Figure 1B,C). We also analyzed the PPI network of these seven genes using STRING (Figure S4). Our risk score formula (Figure 1D) is as follows:

Risk score = MET ∗ 0.327 − DYNLL2 ∗ 0.041 + CDK1 ∗ 0.091 + TNFSF10 ∗ 0.073 − PIP5K1C ∗ 0.428 + MSLN ∗ 0.004 + GKN1 ∗ 0.024(1)

**Figure 2:**
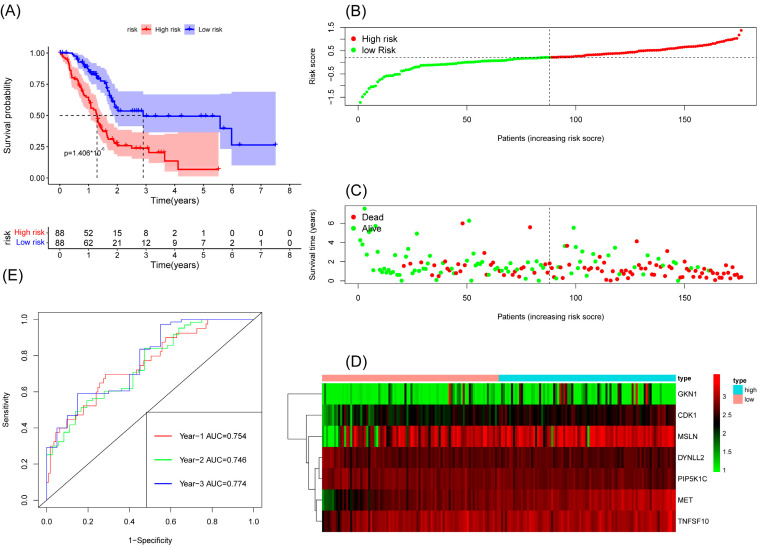
Survival analysis results of a risk-scoring model for TCGA pancreatic adenocarcinoma patients. (**A**) Kaplan–Meier survival analysis of TCGA pancreatic adenocarcinoma patients in high-risk and low-risk groups. (**B**) The overall survival rate and the survival status of TCGA pancreatic adenocarcinoma patients. (**C**) The distribution of risk scores for each patient. (**D**) The expression levels of these 7 anoikis-associated genes in the high-risk and low-risk groups. The green color represents low expression, while the red color represents high expression. (**E**) ROC curve analysis of the risk-scoring model’s 1-year, 2-year, and 3-year OS. OS, overall survival; ROC, receiver operating characteristic.

**Figure 3:**
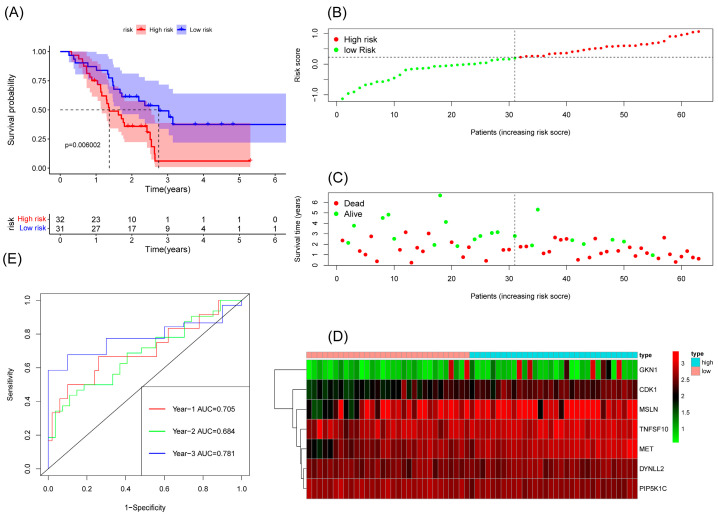
Survival analysis results of a risk-scoring model for GEO pancreatic adenocarcinoma patients. (**A**) Kaplan–Meier survival analysis of GEO pancreatic adenocarcinoma patients in high-risk and low-risk groups. (**B**) The overall survival rate and the survival status of GEO pancreatic adenocarcinoma patients. (**C**) The distribution of risk scores for each patient. (**D**) The expression levels of these 7 anoikis-associated genes in the high-risk and low-risk groups. The green color represents low expression, while the red color represents high expression. (**E**) ROC curve analysis of the risk-scoring model’s 1-year, 3-year, and 5-year OS. OS, overall survival; ROC, receiver operating characteristic.

We constructed a risk score model based on seven anoikis-related genes and divided the patients into high-risk and low-risk groups by the median in the TCGA database. Kaplan–Meier survival analysis displayed that the overall survival time of patients in the high-risk group was significantly lower than that in the low-risk group (Figure 2A). The survival and risk score of each pancreatic adenocarcinoma patient are shown in Figure 2B,C. The gene heat plot shows the expression level of seven genes in each patient (Figure 2D). Finally, we estimated the accuracy of the risk score model by the time ROC curve, and the results displayed that the AUC (area under the curve) values of patients at 1, 2, and 3 years were 0.754, 0.746, and 0.774, respectively (Figure 2E). In the GEO validation cohort, we obtained similar results: the overall survival time of PAAD patients in the low-risk group was longer than that in the high-risk group (Figure 3A–D). The AUC values of 1-, 2-, and 3-year ROC curves of GEO patients were 0.705, 0.684, and 0.781, respectively (Figure 3E).

### 3.2. Immune Infiltration in the Low-Risk and High-Risk Groups

**Figure 4:**
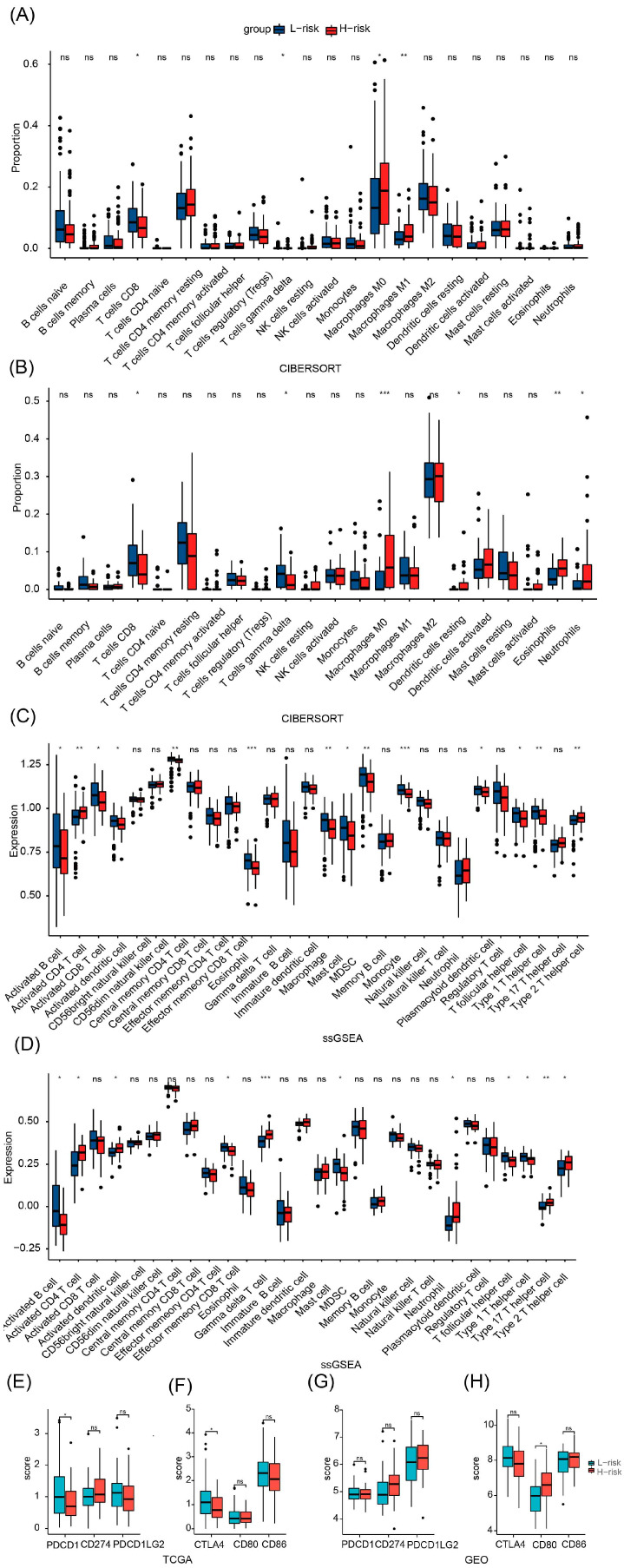
TCGA and GEO tumor microenvironment between high-group and low-risk group. (**A**) Boxplots depicting the CIBERSORT scores of 22 immune cells of the high-risk patients compared to low-risk patients in TCGA. (**B**) Boxplots depicting the CIBERSORT scores of 22 immune cells of the high-risk patients compared to low-risk patients in GEO. (**C**) Boxplots depicting the 28 immune signature ssGSEA scores of the high-risk patients compared to low-risk patients in TCGA. (**D**) Boxplots depicting the 28 immune signature ssGSEA scores of the high-risk patients compared to low-risk patients in GEO. (**E**) The expression of PDCD1, CD274, and PDCD1LG2 was different between high- and low-risk groups in TCGA (**F**) The expression of CTLA4, CD80, and CD86 was different between high- and low-risk groups in TCGA. (**G**) The expression of PDCD1, CD274, and PDCD1LG2 was different between high- and low-risk groups in GEO. (**H**) The expression of CTLA4, CD80, and CD86 was different between high- and low-risk groups in GEO. *, *p* < 0.05, **, *p* < 0.01, ***, *p* < 0.001, ns, no significance.

After dividing the patients into two groups based on risk scores, we compared their immune status. Both in patients with TCGA and GSE57495 pancreatic adenocarcinoma, CIBERSORT analysis showed that compared with the high-risk group, the low-risk group had an increased proportion of T-cells CD8 and a decreased proportion of macrophages M0 (Figure 4A,B). In TCGA, ssGSEA analysis showed that compared with the high-risk group, the low-risk group had an increased content of activated B cells, activated CD8 T cells, activated dendritic cells, central memory CD4 T cells, eosinophils, macrophages, mast cells, MDSCs, monocytes, plasmacytoid dendritic cells, T follicular helper cells, type 1 T helper cells, and type 2 T helper cells, and a decreased content of activated CD4 T cells (Figure 4C). In GSE57495, ssGSEA analysis showed that compared with the high-risk group, the low-risk group had an increased content of activated B cells, activated CD4 T cells, effector memory CD8 T cells, mast cells, MDSCs, T follicular helper cells, and type 1 T helper cells, and a decreased content of activated CD4 T cells, activated dendritic cells, gamma delta T cells, neutrophils, type 17 T helper cells, and type 2 T helper cells (Figure 4D). Finally, compared with the low-risk group, the high-risk group had a decreased expression of the immune checkpoint in TCGA, suggesting that the low-risk group might be suitable for immune checkpoint inhibitor therapy (Figure 4E,F). Unlike TCGA, we did not find a high expression of immune checkpoints in the low-risk group in GSE57495. Instead, the high-risk group overexpressed CD80 (Figure 4G,H).

### 3.3. Analysis of Chemo-Sensitivity in High- and Low-Risk Groups

**Figure 5:**
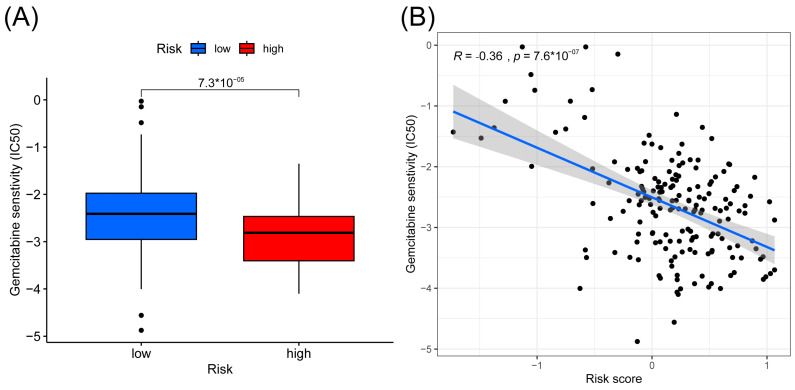
Results of gemcitabine sensitivity analysis. (**A**) Box plots of the estimated IC50 value of gemcitabine between high- and low-risk groups. (**B**) Correlation between IC50 value of gemcitabine and risk score.

Chemo-sensitivity analysis showed that sensitivity to gemcitabine is significantly associated with the risk groups. Patients in the high-risk group were more sensitive to gemcitabine than the low-risk group, with a correlation coefficient of −0.36 between the risk score and IC50 of gemcitabine (Figure 5A,B).

### 3.4. Analysis of Differential Gene Enrichment between High- and Low-Risk Groups

**Figure 6:**
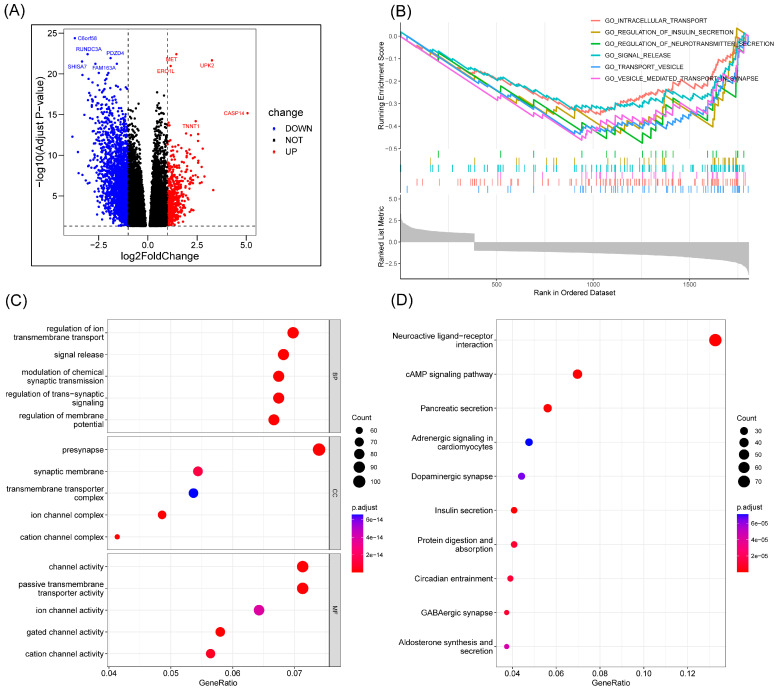
Enrichment analysis. (**A**) Volcano map of differential genes in the high-risk and low-risk groups. (**B**) The important pathway of GSEA analysis. (**C**) The most significant and shared KEGG analysis in the TCGA. (**D**) The most significant and shared GO analysis in the TCGA.

An analysis of the differentially expressed genes (DEGs) of the two risk groups was conducted. Figure 6A shows the volcano map of differentially expressed genes in the high-risk and low-risk groups. GSEA enrichment analysis displayed that these genes were related to intracellular transport, insulin secretion regulation, neurotransmitter secretion, signal release, transport vesicle, and vesicle-mediated transport in synapse (Figure 6B). The GO enrichment analysis showed the involvement of the differential genes in the regulation of ion transmembrane transport, signal release, pre synapse, and channel activity (Figure 6C). KEGG enrichment analysis displayed the relationship between the differential memory and the neuroactive ligand−receptor interaction, cAMP signaling pathway, pancreatic secretion, and other pathways (Figure 6D).

### 3.5. Cluster Analysis and Somatic Mutations Analysis between Different Clusters

**Figure 7:**
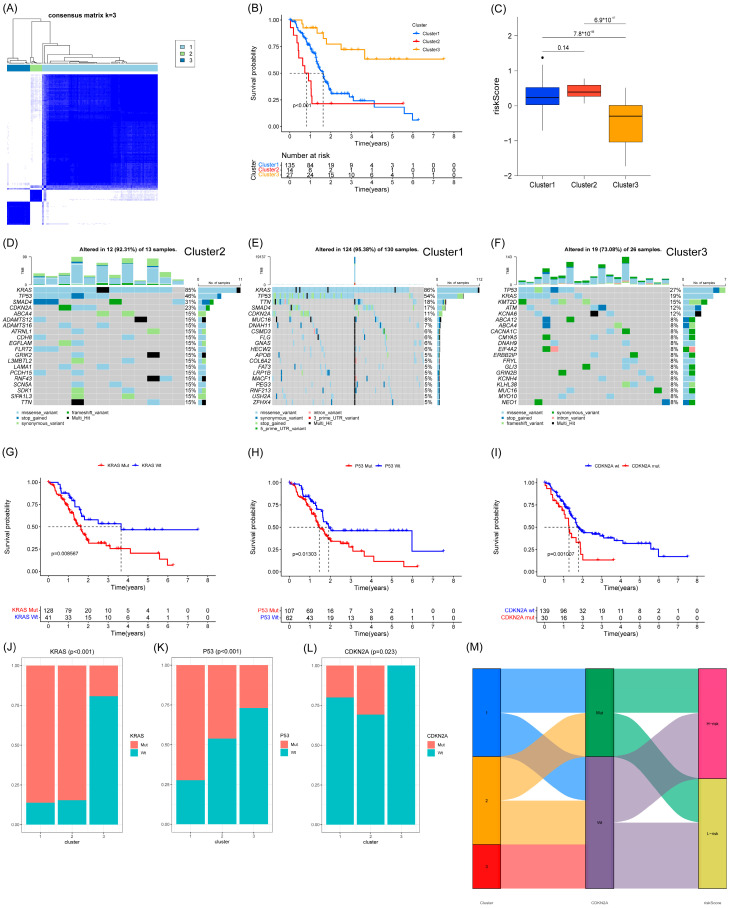
Clustering and mutation analysis based on 7 anoikis-associated genes. (**A**) Three subgroups were identified as having the best values for consensus clustering. (**B**) Survival curves of the 3 subgroups. (**C**) Comparison of risk scores of the 3 subgroups. (**D**–**F**) Mutation status of the 3 subgroups. (**G**–**I**) Survival curves for KRAS, P53, and CDKN2A mutations. (**J**–**L**) Percentage of the 3 mutations in the 3 subgroups. (**M**) Sankey plots for the 3 subgroups, CDKN2A mutations, and high- and low-risk groups.

We performed cluster analysis based on seven prognostic genes and found that they could be clustered into three categories (Figure 7A). Patients in category 3 had the best survival and the lowest risk scores. Patients in category 2 had the worst survival, but the risk scores of patients between categories 1 and 2 statistically showed no difference (Figure 7B,C). We analyzed the somatic mutations in the three categories of patients in order to further investigate the reasons for the differences in survival. The results showed that patients in category 3 with the best survival had significantly fewer KRAS, P53, and CDKN2A mutations (Figure 7D–F). We grouped patients according to the presence of KRAS, P53, and CDKN2A mutations, and Kaplan–Meier survival analysis showed that patients with KRAS, P53, and CDKN2A mutations had worse survival (Figure 7G–I). We show the percentage of KRAS, P53, and CDKN2A mutations in the three categories of patients in a bar chart. The results show that patients in category 3 with the best survival have the lowest percentage of all three mutations, while patients in category 2 with the worst survival have the highest percentage of CDKN2A mutations (Figure 7J–L). Therefore, we speculate that the worst survival in class 2 patients may be associated with CDKN2A mutations, although this may require further confirmation. We drew Sankey plots based on the three clusters of patients, CDKN2A mutation, and high- and low-risk groups, which were used to clearly show the correlation between the three clusters (Figure 7M).

### 3.6. 7 Genes Validation in Single Cell Sequencing

**Figure 8:**
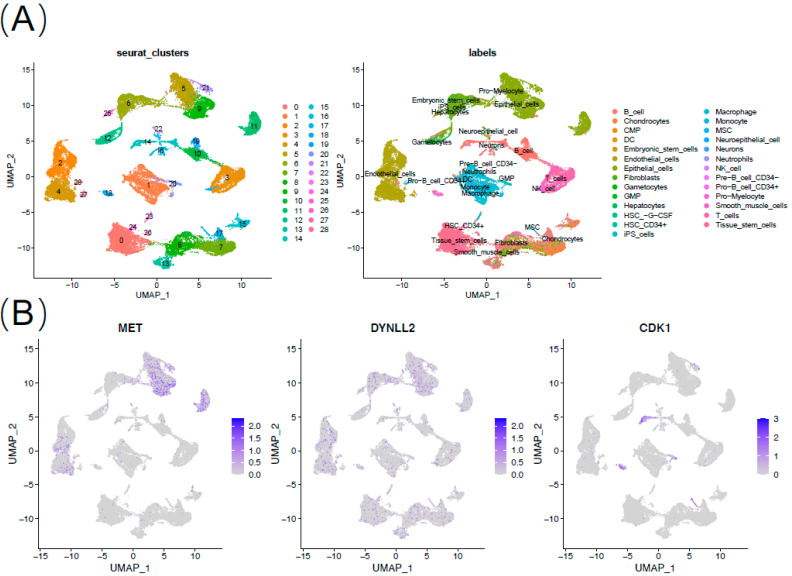

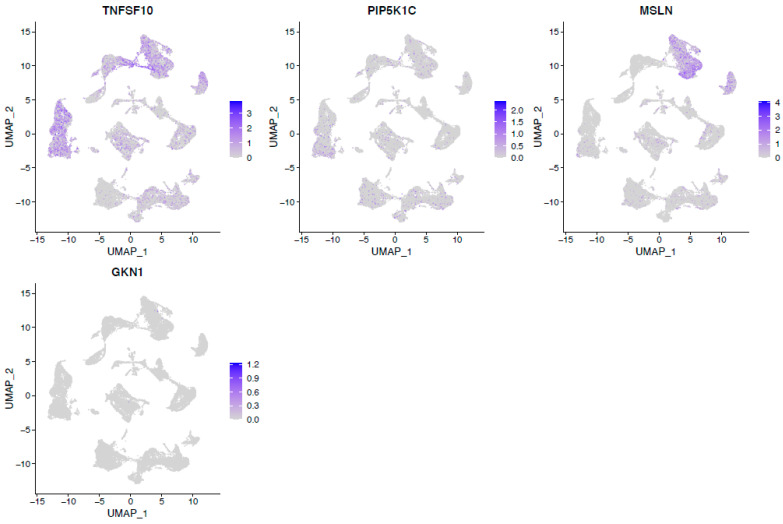
Expression of 7 anoikis-associated genes in single-cell RNA sequencing database. (**A**) Annotation of cell types. (**B**) Expression of MET, DYNLL2, CDK1, TNFSF10, PIP5K1C, MSLN, and GKN1.

To further validate the seven-gene action, we performed analysis using single cell sequencing data. We finally obtained 28 cell clusters and annotated the cell type of each cluster (Figure 8A). MET and TNFSF10 are mainly expressed in epithelial cells and endothelial cells. MSLN is mainly expressed in epithelial cells. CDK1 is mainly expressed in mesenchymal stem cells (MSC) (Figure 8B).

### 3.7. Real-Time Quantitative PCR of Seven Genes in Pancreatic Cancer Cells and Validation in CCLE

**Figure 9:**
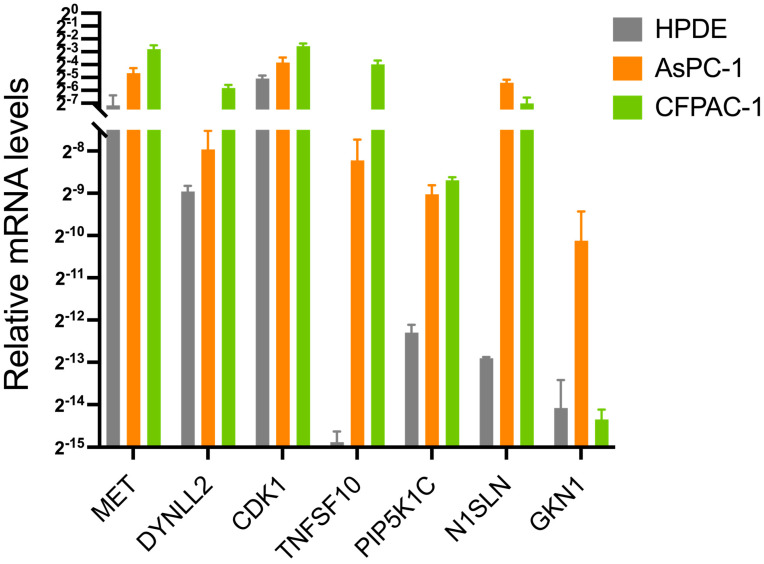
The mRNA expression levels of MET, DYNLL2, CDK1, TNFSF10, PIP5K1C, MSLN, and GKN1 among ASPC-1, CFPAC-1, and HPDE from the in vitro experiments using real-time quantitative PCR.

Expression levels of the anoikis-related genes in normal and pancreatic adenocarcinoma cells were measured. As shown in Figure 9, compared with normal tissues, the expression levels of MET, DYNLL2, CDK1, TNFSF10, PIP5K1C, MSLN, and GKN1 were significantly increased in pancreatic adenocarcinoma cells (*p* < 0.05). The RNA expression levels of these seven genes were further validated in different pancreatic cancer cell lines using Cancer Cell Line Encyclopedia (CCLE) data (Figure S2A–G).

### 3.8. Prognostic Analysis Using Proteomic Data

We performed univariate and multifactorial Cox analyses for all genes available to the proteome. We found significant *p* values for EGFR and MMP2 proteins using univariate and multifactorial Cox analysis (along with risk scores) (Figure S1A,B). Kaplan–Meier survival analysis shows survival grouped by median EGFR and MMP2 protein expression (Figure S1C,D). Interestingly, both EGFR and MMP2 proteins are anoikis-related, which serves as an additional validation of our risk model from the proteomic perspective. This is further evidence that anoikis-related genes have a notable impact on pancreatic adenocarcinoma development.

## 4. Discussion

Anoikis is a crucial defense mechanism of the organism, which plays a pivotal role in preventing the adhesion of detached cells to an erroneous location and restraining their aberrant growth [13]. The disruption of anoikis execution potentially serves as a hallmark of cancer cells and contributes to the progression of tumor invasion, migration, distant organ metastasis, and drug resistance. Presently, it is widely recognized that the occurrence of anoikis apoptosis is contingent upon both the intrinsic and extrinsic pathways [14]. Tumor cells may acquire the ability to evade anoikis and thus enhance their metastatic potential [15].

However, the current literature lacks sufficient investigation into the effects of anoikis-related genes related to the degree of malignancy, immune infiltration, and drug resistance in pancreatic cancer, as well as their prognostic value. Our study aimed to elucidate the pivotal role of anoikis in the development and progression of pancreatic cancer, and its impact on the immune landscape of the pancreatic cancer microenvironment, through a comprehensive analysis of the transcriptome and proteome. We developed a prognostic model incorporating seven key anoikis-related genes, namely MET, DYNLL2, CDK1, TNFSF10, PIP5K1C, MSLN, and GKN1. This model enabled us to stratify patients into high-risk and low-risk subgroups. The MET gene encodes a protein of the receptor tyrosine kinase family. Receptor tyrosine kinases, by binding to ligands of the cell growth factor HGF, transduce signals from the extracellular matrix into the cytoplasm. This, in turn, regulates many physiological processes, including proliferation, scattering, morphogenesis, and survival [16]. There have been many studies demonstrating MET as a poor prognostic gene for pancreatic adenocarcinoma, which is consistent with our findings [17,18]. The CDK1 gene expresses cyclin-dependent kinase 1, a highly conserved protein kinase complex that is essential for the G1/S and G2/M phase transitions of the eukaryotic cell cycle [19]. Mitotic cell cycle proteins stably bind to this protein and function as regulatory subunits. Phosphorylation and dephosphorylation of this protein also play an important regulatory role in cell cycle control [20]. Wijnen and colleagues also confirmed that CDK1 is a prognostic risk gene for pancreatic adenocarcinoma and suggested that the inhibition of CDK1 could be a new strategy for the treatment of pancreatic adenocarcinoma [21]. TNFSF10 encodes a protein that is a cytokine belonging to the tumor necrosis factor (TNF) ligand family. This protein preferentially induces apoptosis in transformed and tumor cells, but does not appear to kill normal cells [22]. At present, there is no article about the relationship between the TNFSF10 gene and the prognosis of pancreatic adenocarcinoma. Our study is the first to confirm that TNFSF10 is a prognostic risk gene for pancreatic adenocarcinoma. The MSLN gene encodes a pre-protein, which can be hydrolyzed to produce two protein products, megakaryocyte potential factor and mesodermin. As a kind of cytokine, megakaryocyte augmenter can stimulate the colony formation of bone marrow megakaryocytes. Mesothelin is a cell surface protein anchored by glycosyl phosphatidylinositol, which can play a role as cell adhesion protein [23]. GKN1 gene expresses gastric factor 1, which may be involved in maintaining the integrity of the gastric mucosal epithelium [24]. At present, there is no report that MSLN and GKN1 are prognostic risk genes for pancreatic adenocarcinoma. The expressed protein of the DYNLL2 gene is one of the non-catalytic auxiliary components of the cytoplasmic kinesin 1 complex [25]. This cofactor component is thought to have a role in linking kinesin to cargo. Cytoplasmic kinesin 1 acts as a motor for the retrograde movement of vesicles and organelles along microtubules within the cell, and may play a role in altering or maintaining the spatial distribution of cytoskeletal structures [25]. PIP5K1C encodes type I phosphatidylinositol 4-phosphate 5-kinase. The encoded protein catalyzes the phosphorylation of phosphatidylinositol 4-phosphate to produce phosphatidylinositol 4,5-diphosphate. This enzyme exists in synapses and is found to play a role in endocytosis and cell migration [26]. No study has confirmed that DYNLL2 and PIP5K1C are prognostic protective gene for pancreatic adenocarcinoma, and this study is the first to do so. This also provides new targets for molecular biology research and the potential treatment of pancreatic adenocarcinoma.

With these seven-gene signatures in the risk model, we conducted a comprehensive analysis and assessment of the two subgroups, which revealed significant differences in the survival time of patients with pancreatic cancer between the high-risk and low-risk subgroups. Furthermore, we investigated immune cell infiltration and immune targets to identify differences between the subgroups. Moreover, we discussed the potential of immunotherapy in pancreatic cancer based on a risk score model. Given that the presence of anoikis-related genes has been found to be closely associated with chemo-resistance in various tumors, we also explored the impact of this model on gemcitabine resistance.

Additionally, we observed an inverse correlation between gemcitabine sensitivity and risk score, indicating that patients with higher risk scores were more likely to exhibit gemcitabine resistance. Our findings suggest that patients with higher risk scores might benefit more from immune checkpoint inhibitors than from traditional gemcitabine-based treatment regimens. Based on the seven risk score genes, we stratified the pancreatic cancer patients into tree clusters. Interestingly, TP53, KARS, and CDKN2A had higher mutations in all three groups and all three genes were associated with prognosis, which again validated the existing findings [27].

The mutations of Kras, Tp53, and CDKN2A have been extensively investigated and consistently shown to exert a profound impact on the survival of pancreatic cancer patients, manifesting in both early and recent clinical advancements. Notably, these mutations were found to be enriched across all three of our study cohorts, further underscoring their clinical significance. As a pivotal initiator of exocrine tumorigenesis, the Kras gene has been shown through multiple investigations to harbor mutations in the majority of low-grade pancreatic intraepithelial neoplasia (PanIN) and intraductal papillary mucinous neoplasm (IPMN) lesions. Such findings underscore the fundamental role of Kras mutations in the early stages of pancreatic cancer development [28,29]. Using the KPC-GEMM model, investigators have demonstrated that the withdrawal of the oncogenic Kras gene results in the regression of invasive tumors, including metastatic lesions. These findings suggest that Kras may represent a promising therapeutic target for the treatment of pancreatic cancer [30,31,32]. Remarkably, inducible systems have revealed that pancreatic intraepithelial neoplasia (PanIN) lesions can undergo redifferentiation into acinar cells in the absence of tumor suppressor gene loss. However, in preclinical models, the withdrawal of mutant Ras signaling, accompanied by the loss of tumor suppressive functions, has been shown to promote tumor recurrence through Ras-independent mechanisms. These findings have important implications for the development of effective therapeutic strategies against pancreatic cancer [33]. These observations highlight the crucial role of TP53 in the pathogenesis and progression of PDAC, and underscore its potential as a therapeutic target in this deadly disease. TP53 stands out as the most commonly mutated tumor suppressor gene in pancreatic ductal adenocarcinoma (PDAC), and its functional changes have been linked to various oncogenic outcomes, encompassing heightened genomic instability [33], cellular metabolism reprogramming [34], and augmented metastatic propensity [35]. CDKN2A is frequently inactivated by homozygous deletion at the chromosomal 9p21 locus, often co-occurring with the loss of an adjacent interferon cluster in 50% of cases. This interruption of interferon signaling leads to a “cold” tumor immune microenvironment and resistance to immune-based therapies [36], thereby contributing to adverse patient outcomes. These findings highlight the clinical significance of CDKN2A alterations in pancreatic cancer and underscore the potential of interferon-based therapies as a promising treatment approach.

The investigation of differential expression patterns of anoikis-related genes in pancreatic cancer not only enhances our understanding of the refractory nature of this disease, but also contributes to the development of additional personalized and precise immunotherapy regimens. Given that pancreatic cancer is considered an immunologically “cold” tumor, the tumor microenvironment and immune cell infiltration are inadequately explored, making it challenging to predict patient responsiveness to immunotherapy. Many immunotherapy strategies that have demonstrated promising results in preclinical studies have failed to yield convincing outcomes in clinical trials, underscoring the limitations and insufficiencies of current preclinical models for pancreatic cancer. We evaluated the utility of risk scores in predicting the response of pancreatic cancer to immunotherapy, and analyzed disparities in the expression of immune-associated cell tumors between high- and low-risk scores. Therefore, our model may enable the prediction of patients’ susceptibility to immune checkpoint inhibitors based on anoikis, and facilitate the prediction of treatment efficacy, thus providing novel insights into the immunotherapy of pancreatic cancer.

Furthermore, we analyzed single-cell sequencing data pertaining to the seven molecules included in our model, and observed distinct distributions of positive cells expressing these molecules in pancreatic cancer. Notably, these cells were primarily epithelial cells, endothelial cells, and partly immune progenitor cells. Considering the impact of the risk model on immune infiltration, we suggest that the anti-anoikis characteristics of pancreatic cancer cells may contribute to shaping the immune suppressive microenvironment of pancreatic cancer, ultimately resulting in its ‘immune desert’-like appearance.

## 5. Conclusions

Our comprehensive investigation utilizing multi-omics verification techniques has revealed the significance of anoikis-related genes in predicting the prognosis of pancreatic adenocarcinoma. Through our study, we have identified a prognostic model based on seven genes, demonstrating strong predictive performance. Furthermore, we have elucidated the impact of KRAS, P53, and CDKN2A mutations on pancreatic adenocarcinoma prognosis, with CDKN2A mutation serving as a potential prognostic factor independent of anoikis. Our risk score suggests that gemcitabine may be a vital therapeutic agent for high-risk patients. Taken together, our findings underscore the pivotal role of anoikis in the development of pancreatic adenocarcinoma, and provide novel targets for researchers to explore the mechanism of action of anoikis, and for clinicians to apply the potential treatments to pancreatic adenocarcinoma patients.

## Data Availability

The data supporting this study’s findings are openly available in the Therapeutically Applicable Research to TCGA and GEO (GENE EXPRESSION OMNIBUS).

## Supplementary Materials

The following supporting information can be downloaded at: https://www.mdpi.com/article/10.3390/cancers15123146/s1, Figure S1: Proteomics of TCGA pancreatic adenocarcinoma; Figure S2: CCLE analysis to model gene expression; Figure S3: Flow Chart; Figure S4: PPI; Table S1: Primer sequences for RT-qPCR.
