# Supplementary material for: Anoikis-Related Gene Signature for Prognostication of Pancreatic Adenocarcinoma: A Multi-Omics Exploration and Verification Study
Source: Cancers (Basel). 2023 Jun 11;15(12):3146. doi: 10.3390/cancers15123146 (PMC10296373; doi:10.3390/cancers15123146)
Supplement: Supplementary file 1 [file cancers-15-03146-s001.zip › Table S1.docx]

**Table S1 Primer sequences for RT-qPCR**

| Genes | Forward | Reverse |
| --- | --- | --- |
| MET | AGCAATGGGGAGTGTAAAGAGG | CCCAGTCTTGTACTCAGCAAC |
| DYNLL2 | ACCCTACCTGGCATTGTATCG | AGCCTGACTTGAAGAGGAGGA |
| CDK1 | AAACTACAGGTCAAGTGGTAGCC | TCCTGCATAAGCACATCCTGA |
| TNFSF10  PIP5K1C  MSLN  GKN1 | TGCGTGCTGATCGTGATCTTC  AGACCGTCATGCACAAGGAG  CCCATTGGACCTGCTGCTATT  CTGTCCACTGCTTTCGTGAAG | GCTCGTTGGTAAAGTACACGTA  CAGTACAGCCCATAGAACTTGG  CATTGGCCTTCGTGATGCG  GTCCCATCCGTTGTTATTGTCAA |
