# Supplementary figures and images for: Anoikis-Related Gene Signature for Prognostication of Pancreatic Adenocarcinoma: A Multi-Omics Exploration and Verification Study
Source: Cancers (Basel). 2023 Jun 11;15(12):3146. doi: 10.3390/cancers15123146 (PMC10296373; doi:10.3390/cancers15123146)

(A)

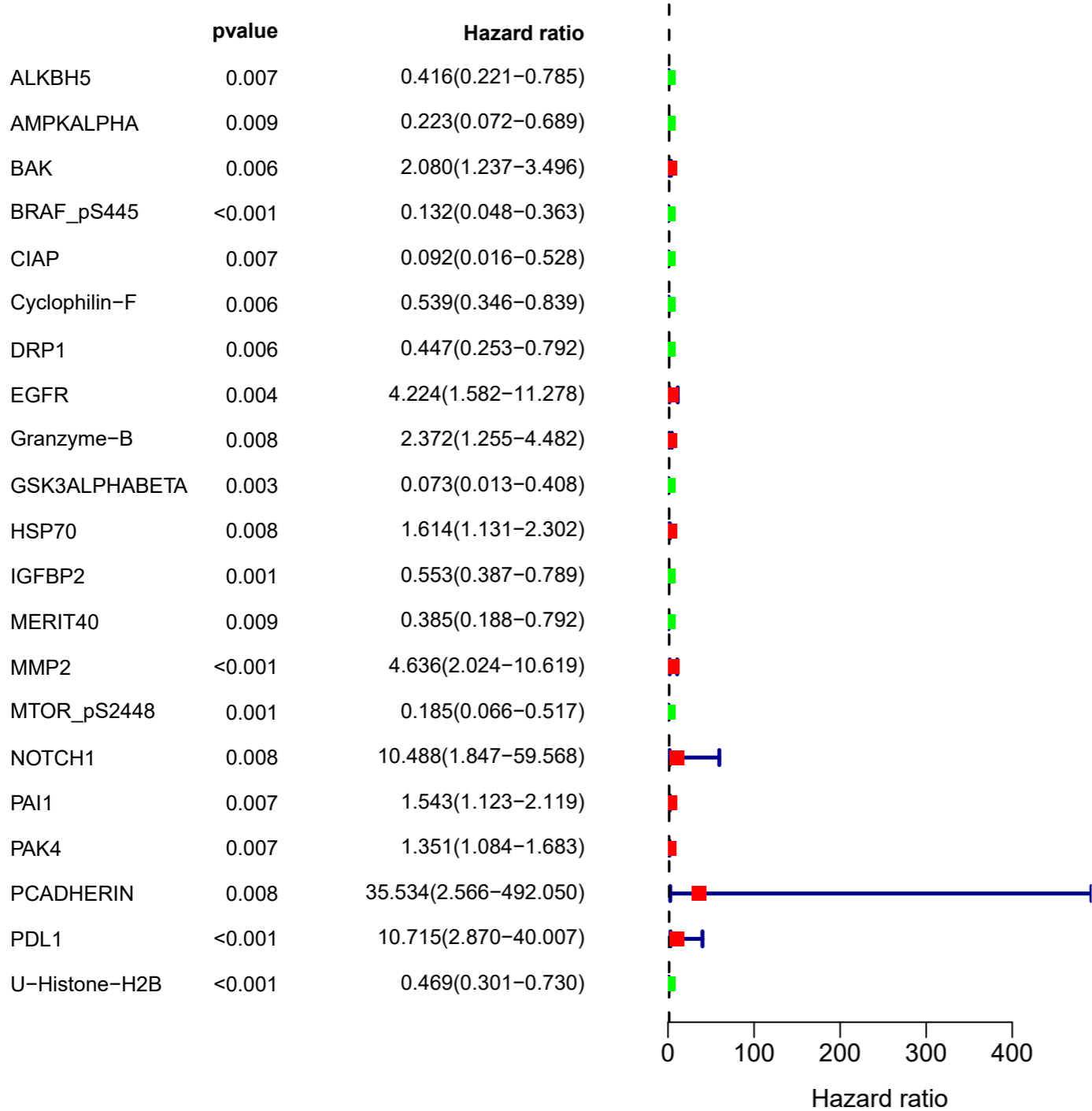

(B)

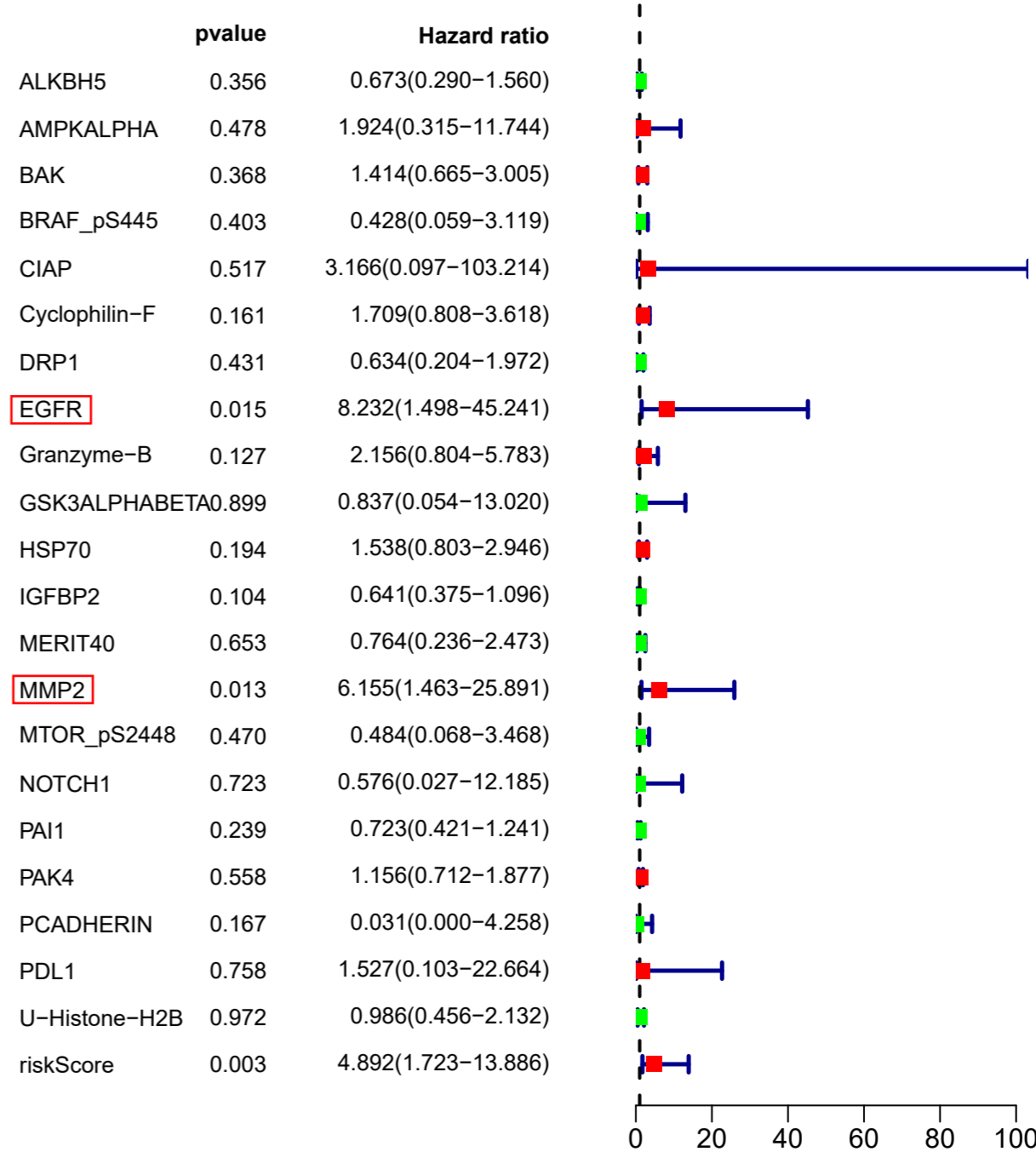

(C)

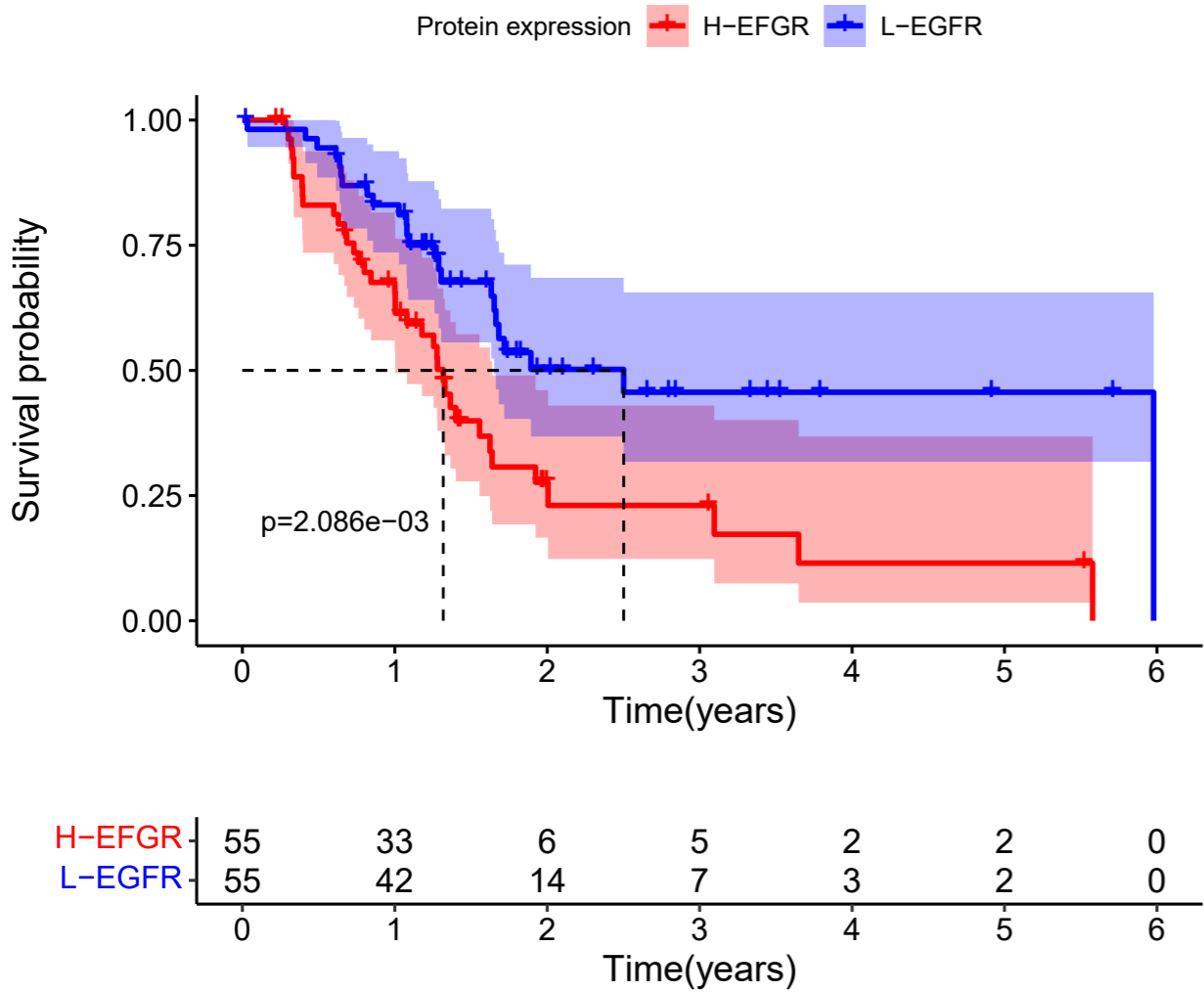

(D)

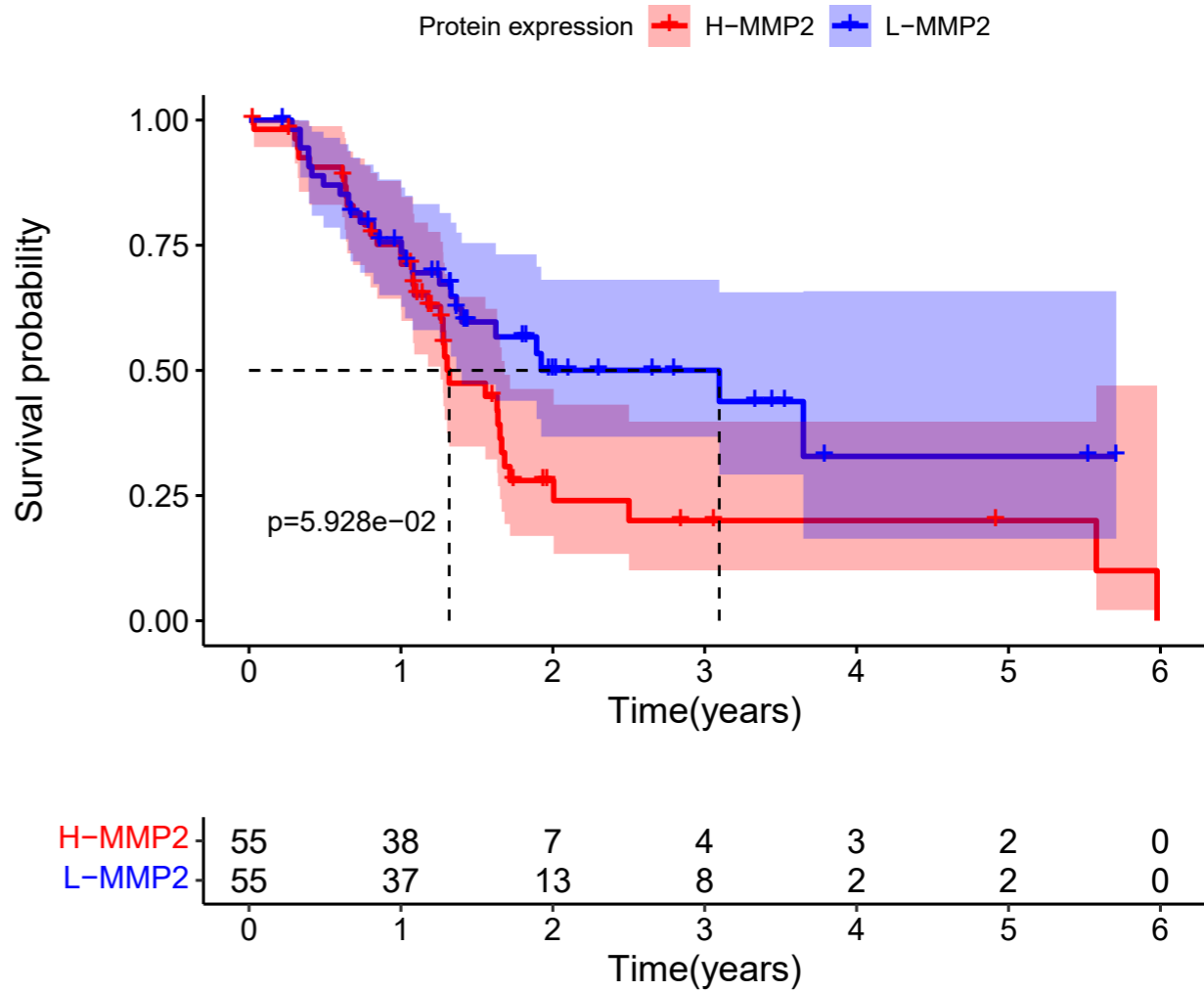

Supplement: Supplementary file 1 [file cancers-15-03146-s001.zip › Figure S1-Proteomics of TCGA pancreatic adenocarcinoma.pdf]

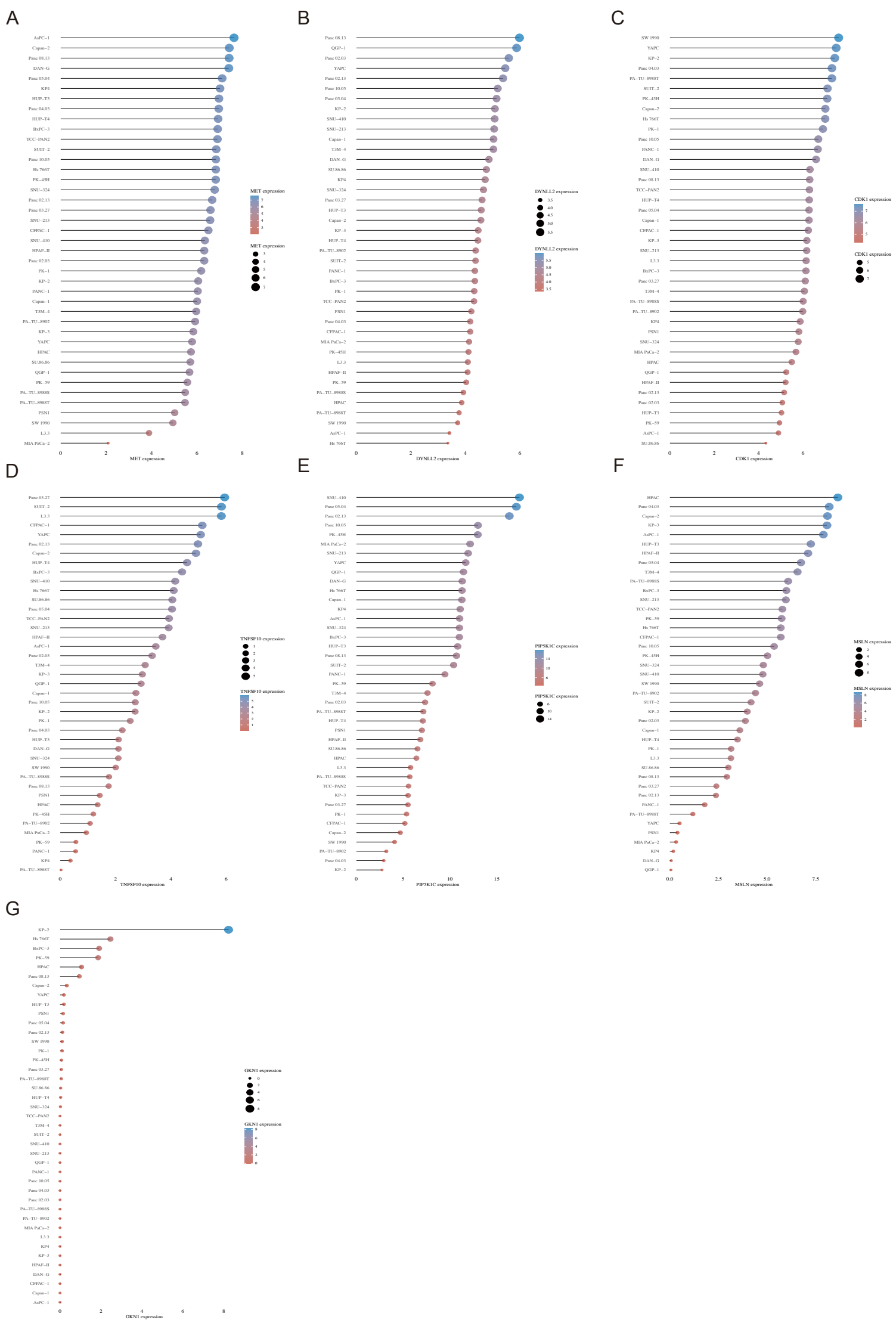

Supplement: Supplementary file 1 [file cancers-15-03146-s001.zip › Figure S2.pdf]

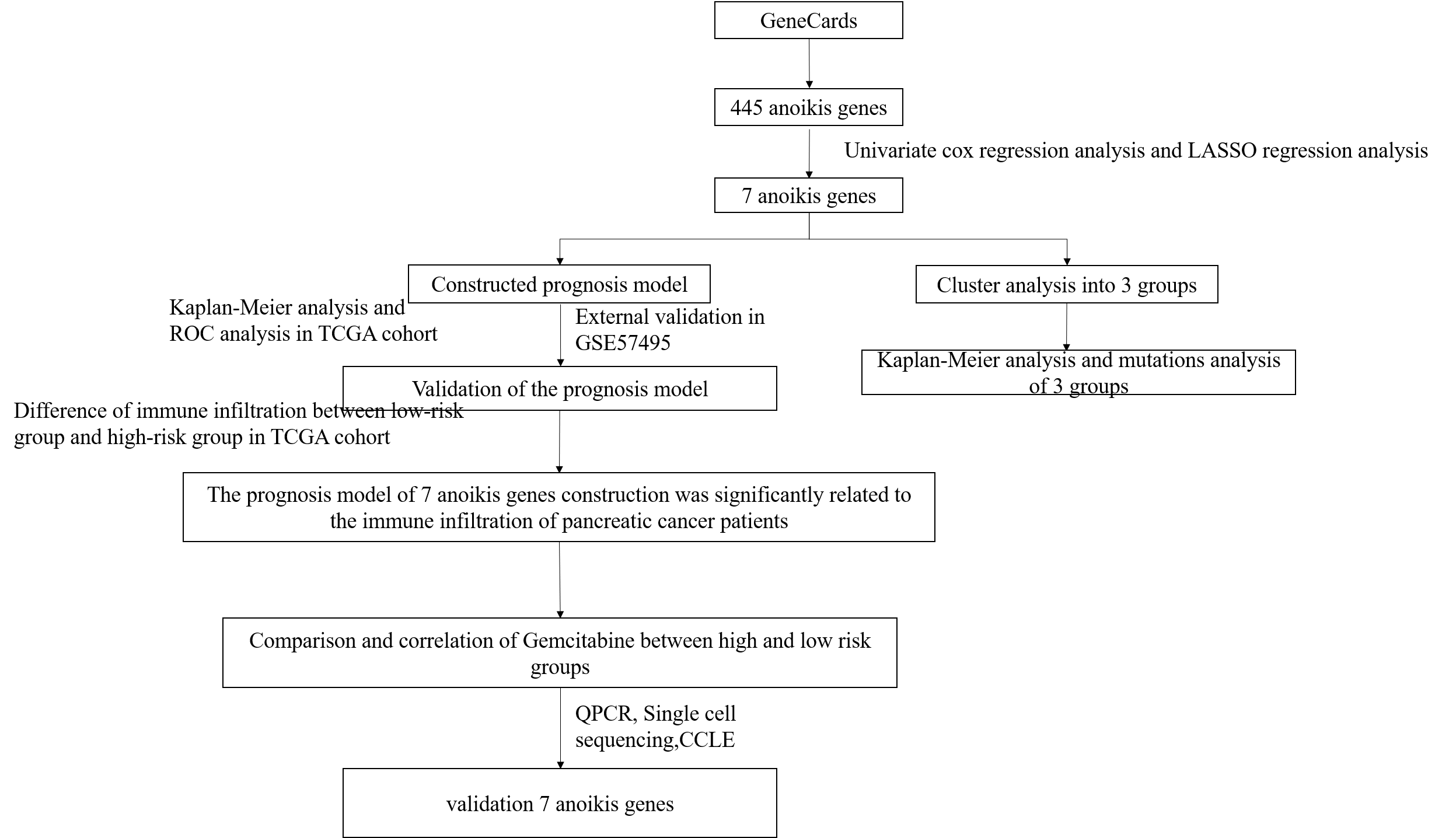

Supplement: Supplementary file 1 [file cancers-15-03146-s001.zip › Figure S3.tif]

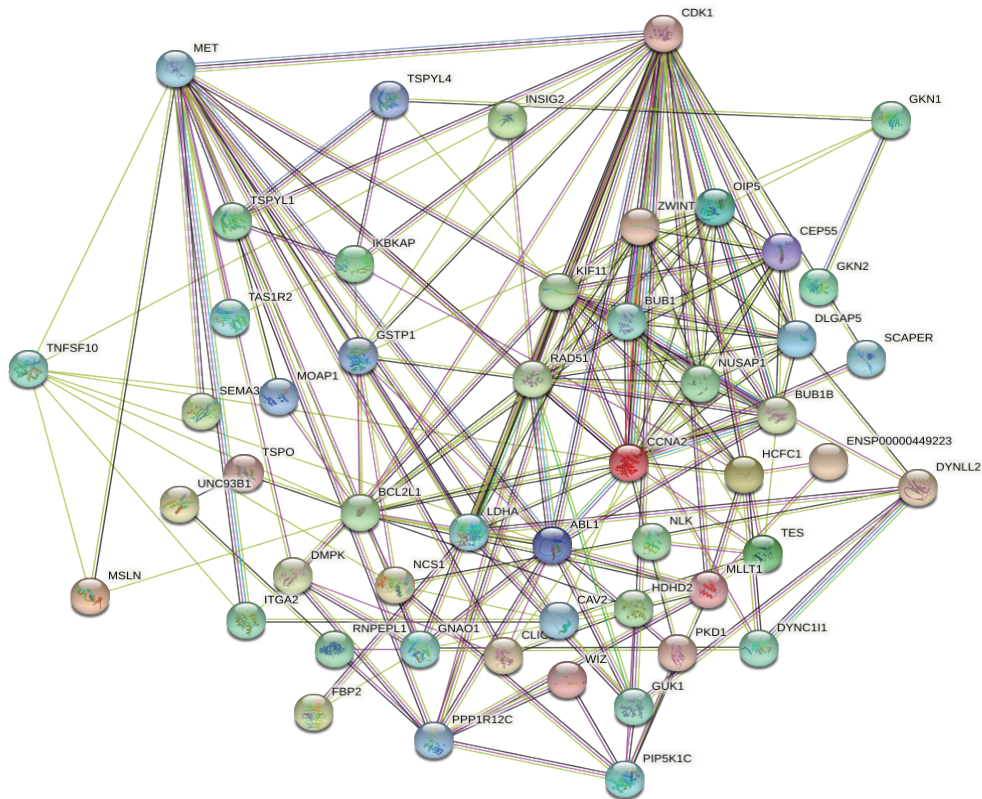

Supplement: Supplementary file 1 [file cancers-15-03146-s001.zip › Figure S4.pdf]
